# The impact of hypertensive disorders during pregnancy on maternal perinatal depressive and anxiety symptoms

**DOI:** 10.1192/j.eurpsy.2021.483

**Published:** 2021-08-13

**Authors:** B. Dachew, R. Alati

**Affiliations:** School Of Public Health, Curtin University, Perth, Australia

**Keywords:** ALSPAC, anxiety symptoms, depressive symptoms, Gestational hypertension

## Abstract

**Introduction:**

Existing evidence regarding the association between hypertensive disorders of pregnancy (HDP) and the risk of maternal mental illness is inconclusive.

**Objectives:**

This study aimed (i) to investigate the relationship between HDP (pre-eclampsia and gestational hypertension) and the risk of depressive and anxiety symptoms during pregnancy and in the postpartum period and (ii) to test whether parity moderates the association between HDP and antenatal and postnatal anxiety and depressive symptoms.

**Methods:**

The study cohort consisted of more than 8500 mothers who participated in the Avon Longitudinal Study of Parents and Children (ALSPAC), UK. Maternal antenatal and postnatal depressive and anxiety symptoms were assessed using the Edinburgh Postnatal Depression Scale (EPDS) and the Crown-Crisp Experiential Index (CCEI), respectively. Univariable and multivariable logistic and linear regression analyses were used to examine the associations.

**Results:**

Mothers with pre-eclampsia had a 53% (aOR= 1.53; 95% CI, 1.06-2.23) increased risk of antenatal depressive symptoms compared with those without pre-eclampsia. Having pre-eclampsia and being a nulliparous woman resulted in a 2.75 fold increased risk of antenatal depressive symptoms (p-value for interaction = 0.03). Gestational hypertension was associated with antenatal depressive and anxiety symptoms. We found no associations between pre-eclampsia and/or gestational hypertension and postnatal anxiety and depressive symptoms.

**Conclusions:**

Our study showed that mothers with HDP were at higher risk of antenatal depressive and anxiety symptoms. Nulliparous women with pre-eclampsia are a higher risk group for depression during pregnancy.

**Disclosure:**

No significant relationships.

